# Multisensory synchrony of contextual boundaries affects temporal order memory, but not encoding or recognition

**DOI:** 10.1007/s00426-022-01682-y

**Published:** 2022-04-28

**Authors:** Vincent van de Ven, Guyon Kleuters, Joey Stuiver

**Affiliations:** 1 Department of Cognitive Neuroscience, Faculty of Psychology and Neuroscience, Maastricht University, P.O. Box 616, 6200 MD Maastricht, The Netherlands; 2 Faculty of Health, Medicine and Life Sciences, Maastricht University, Maastricht, The Netherlands

## Abstract

We memorize our daily life experiences, which are often multisensory in nature, by segmenting them into distinct event models, in accordance with perceived contextual or situational changes. However, very little is known about how multisensory boundaries affect segmentation, as most studies have focused on unisensory (visual or audio) segmentation. In three experiments, we investigated the effect of multisensory boundaries on segmentation in memory and perception. In Experiment 1, participants encoded lists of pictures while audio and visual contexts changed synchronously or asynchronously. After each list, we tested recognition and temporal associative memory for pictures that were encoded in the same audio-visual context or that crossed a synchronous or an asynchronous multisensory change. We found no effect of multisensory synchrony for recognition memory: synchronous and asynchronous changes similarly impaired recognition for pictures encoded at those changes, compared to pictures encoded further away from those changes. Multisensory synchrony did affect temporal associative memory, which was worse for pictures encoded at synchronous than at asynchronous changes. Follow up experiments showed that this effect was not due to the higher dimensionality of multisensory over unisensory contexts (Experiment 2), nor that it was due to the temporal unpredictability of contextual changes inherent to Experiment 1 (Experiment 3). We argue that participants formed situational expectations through multisensory synchronicity, such that synchronous multisensory changes deviated more strongly from those expectations than asynchronous changes. We discuss our findings in light of supportive and conflicting findings of uni- and multi-sensory segmentation.

## Introduction

We temporally segment our everyday experiences into distinct episodic events in memory (Brunec et al., 2018; Radvansky & Zacks, 2017; Zacks, 2020). Segmentation crucially depends on perceived changes in contextual features, which constitute deviations from situational predictions and thereby act as event boundaries separating previous from current event models in working memory (Radvansky & Zacks, 2017; Richmond & Zacks, 2017). Temporal segmentation occurs in many different situations, from crossing a doorway segmenting experiences to different spatial environments (Radvansky & Copeland, 2006; van Helvoort et al., 2020) to narrative changes in space, time, perspective or action goals segmenting our memory of film or story materials (Newtson, 1973; Radvansky & Copeland, 2010; Schwan & Garsoffky, 2004; Schwan et al., 2000; Swallow et al., 2018; Zacks et al., 2009). How we segment our experiences influences how we perceive and remember them. The detection and processing of contextual boundaries comes at an attentional cost (Huff et al., 2012), but can also enhance recognition memory for information presented near those boundaries relative to information away from boundaries (Aly & Turk-Browne, 2016; Newtson & Engquist, 1976; Swallow et al., 2009). Further, experiences from the same situational context become more strongly associated in memory, such that experiences sharing a common contextual segment in memory can be more readily or accurately retrieved than experiences from different contextual segments (Radvansky & Copeland, 2006; Smith, 1985; van Helvoort et al., 2020). As such, boundaries help shape memory and understanding of temporally structured experiences.

Segmentation has often been studied using movie clips, in which the contextual changes are typically multisensory (i.e., audio and visual) (Baldassano et al., 2017; Ben-Yakov & Henson, 2018; Boltz, 1992; Chen et al., 2017; Cutting, 2019; Furman et al., 2007; Huff et al., 2014; Newtson, 1973; Schwan & Garsoffky, 2004; Schwan et al., 2000; Zacks et al., 2009). Segmentation studies that used unisensory contextual features showed comparable segmentation effects in the visual (Ezzyat & Davachi, 2011; Newberry & Bailey, 2019; Zacks et al., 2009) and auditory domain (Baldassano et al., 2018; Huff et al., 2018; Sridharan et al., 2007), suggesting that segmentation is independent of sensory modality. However, the contribution of multisensory dimensionality to temporal segmentation has remained under-investigated. Recently, Meitz et al. (Meitz et al., 2020) compared the detection of film cuts occurring between or within scenes (i.e., at or away from filmic boundaries) when movie clips were presented with or without their audio tracks. They found that participants better detected film cuts at boundaries than cuts away from boundaries, irrespective of the audibility of the audio track. Likewise, recognition memory was better for between-scene changes than for within-scene changes, irrespective of whether the audio track was played during encoding. The authors suggested that segmentation followed semantically congruent boundaries, rather than multisensory complexity or integration. Possibly, the semantic associations between the audio and visual tracks made the multisensory information redundant in segmenting perception and memory. This suggestion is in line with a previous finding (Meyerhoff & Huff, 2016) that reversing the visual track of movie clips did not decrease subsequent recognition performance compared to synchronous audio and visual tracks, indicating that event memory depended on semantic rather than multisensory congruency.

The lack of a multisensory effect in segmentation appears at odds with observations that boundary detection increases with increasing number of changing narrative dimensions, such as space, time or action goal (Zacks et al., 2009). Event segmentation theories postulate that more concurrently changing contextual features would constitute a larger deviation of situational predictions in working memory (Zacks, 2020). Likewise, an effect of audio-visual integration on boundary processing would be expected from the perspective that multisensory synchronization facilitates stimulus encoding (Chen & Spence, 2010; ten Oever et al., 2013) and memory formation of individual items (Botta et al., 2011; Thompson & Paivio, 1994). Indeed, audio-visually presented movie clips are subsequently better recognized than audio-only or visual-only clips (Meyerhoff & Huff, 2016). Further, visual (or auditory) information is better encoded when semantically congruent auditory (resp., visual) information is presented synchronously rather than asynchronously with the other modality (Bushara et al., 2003; Miller & D’Esposito, 2005; Van Atteveldt et al., 2007; Chen & Spence, 2010; ten Oever et al., 2013).

Another point of contention is that the (lack of) effect of multisensory boundaries in previous studies was obtained from recognition memory, rather than associative memory. The enhanced recognition of items encoded at contextual boundaries (as opposed to those away from boundaries) may come with the trade-off of impaired temporal binding between items crossing a boundary in associative memory (DuBrow & Davachi, 2013; Heusser et al., 2018; van de Ven et al., 2021). The context dependence of associative memory could thus be more sensitive than recognition memory to the effect of multisensory boundary processing and segmentation (Clewett & Davachi, 2017), but this scenario remains to be tested.

To investigate these issues, we conducted three experiments in which participants encoded lists of random, unrelated visual objects while audio and/or visual contextual features changed after a number of objects. Previous studies using this design showed that visual (DuBrow & Davachi, 2013, 2014; Heusser et al., 2018) or temporal boundaries (van de Ven et al., 2021) impaired temporal order memory judgments for picture pairs crossing a boundary during encoding relative to temporal memory judgments for picture pairs coming from the same context. The contextual changes thus mimicked the effect of boundaries in narrative segmentation (Ezzyat & Davachi, 2011; Lositsky et al., 2016). In our experiments, we used continuously presented audio and visual contexts in the form of, respectively, ambient soundscapes and colored frames. The audio and visual contexts were not semantically related, and neither were the pictures semantically related to the audio or visual contexts. In Experiment 1, we manipulated the synchrony of audio-visual boundaries and assessed its effect on temporal order memory performance. We hypothesized that, if multisensory synchronicity affects segmentation, then temporal order memory performance for items crossing a synchronous multisensory boundary would be worse than performance for items crossing an asynchronous multisensory boundary. In this experiment, the contexts were continuously multisensory. The perceptual expectations would therefore likely differ from a unisensory context, such that an asynchronous multisensory boundary (e.g., changing audio but continuous visual context) may affect segmentation differently than a unisensory boundary (changing audio in the absence a visual context). If boundary processing is sensitive to perceptual dimensionality, then multisensory boundaries would impair across-context temporal order judgments more than unisensory boundaries. We tested this hypothesis in Experiment 2. Finally, the mix of synchronous and asynchrous boundaries in Experiment 1 could be experienced as irregular or unpredictable, and thereby affect boundary processing independently of perceptual dimensionality. We investigated this issue in Experiment 3, in which we manipulated temporal expectancy of unisensory contextual changes, such that audio or visual changes occurred at regular or irregular intervals. If boundary processing depends on temporal expectations about when a boundary occurs, then across-context temporal order memory judgments would be worse for irregularly than regularly distributed boundaries.

## Experiment 1

In Experiment 1, we tested the hypothesis that synchronous multisensory boundaries would impair temporal order processing in memory more than asynchronous boundaries. To this end, we manipulated the synchronicity of multisensory contextual boundaries during the encoding of a series of visual objects and assessed its effect on subsequent recognition or temporal memory performance. While audio and visual contexts were continuously and concurrently presented, the contextual changes occurred in synchrony (multisensory audio and visual context change) or out of synchrony (unisensory change of either audio or visual context).

## Methods

### Participants

We initially recruited 34 participants in the age range of 18–40 years from the academic environment of Maastricht University. Participants were recruited via social media platforms and were required to have at-home access to a computer or laptop, headphones, Internet access to download and install the experiment software and a quiet place without distractions (see below for further details). Participants who could not or were not willing to install the experiment software were excluded from participation in the study. Of the recruited sample, 24 participants (16 females; mean ± SD age = 21.2 ± 2.0 years, range 18 to 27) successfully installed and completed the experiment (the other 10 participants could not install, run or complete the experiment for technical reasons). All participants provided informed consent before participating in the experiment and were monetarily compensated. The study was approved by the ethical committee of the Faculty of Psychology and Neuroscience of Maastricht University.

### Procedure

Due to national regulations in response to the COVID-19 pandemic during 2020–2021 in The Netherlands, we designed the experiment so that it could be completed at home. We programmed the experiment in Psychopy (Peirce, 2007), which has been shown to operate reliably at high temporal precision and with limited variations across operating systems (Bridges et al., 2020; Garaizar & Vadillo, 2014). We asked participants to download and install the latest version of Psychopy from the website. After successful installation, participants downloaded, unpacked and ran the experiment code. Participants were instructed to use headphones in order to maximize audibility of the sound stimuli and minimize distracting environmental sounds. Participants were further instructed to reduce distractions to a minimum by turning their mobile phone or social media apps off during the experiment. Prior to starting the experiment, participants were contacted via online conference call by one of us (JS or GK) to verify correct software installation and compliance to task instructions. After completion of the experiment, participants were asked to return the data files by email to the investigators.

### Materials and task design

**Fig. 1:**
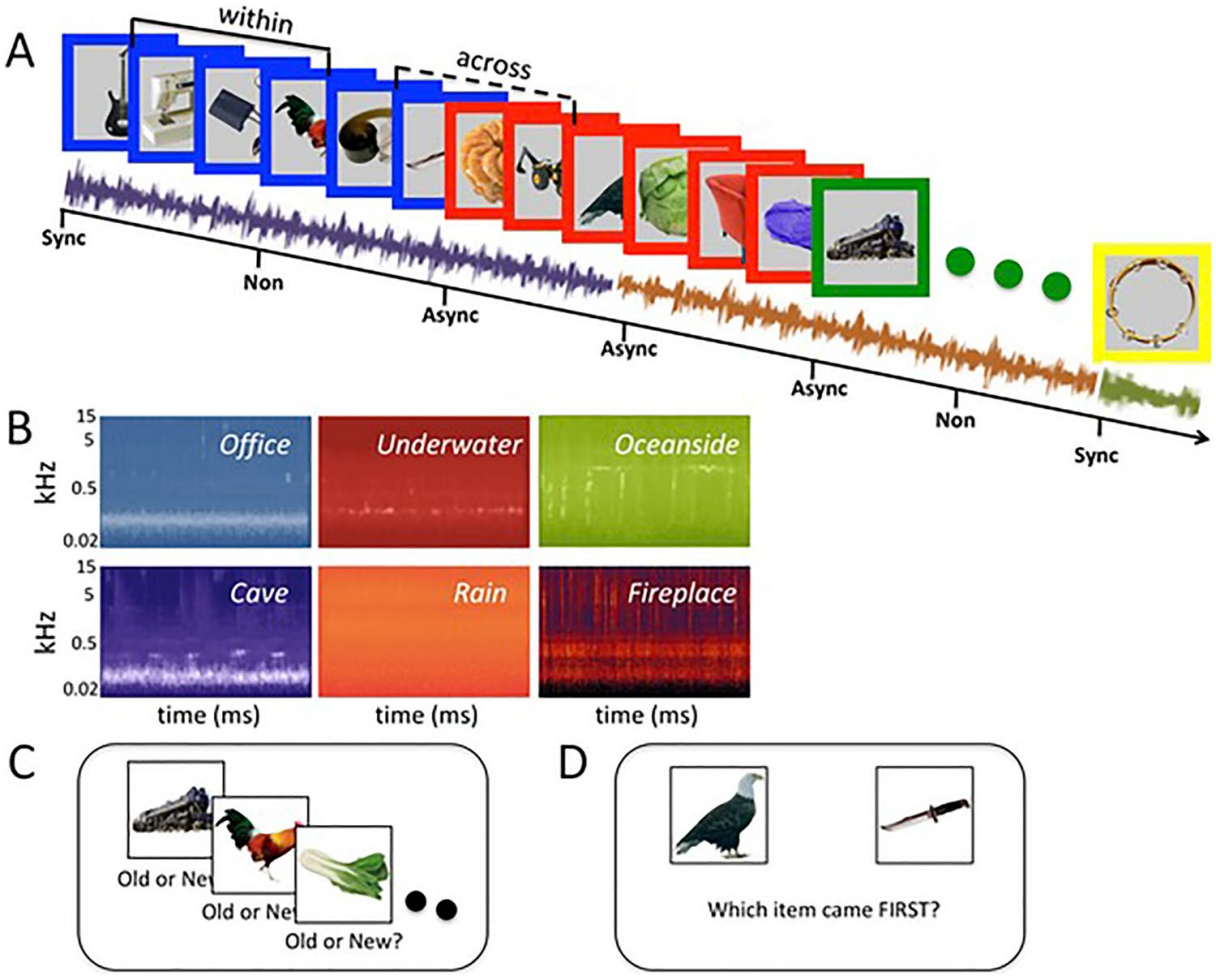
Experimental design. **A** In Experiment 1, lists of pictures are shown on an audiovisual background of audio soundscapes and colored frames. In half of the lists, frame color changed every six pictures while soundscapes changed every nine pictures (vice versa for other half of lists), such that audio and visual contexts sometimes changed synchronously (Sync) or asynchronously (Async). After each list, recognition memory for boundary (Sync or Async) and non-boundary items (Non) was tested, as well as temporal order memory for items drawn from the same audiovisual contexts (*Within*) or crossing a Sync or Async boundary (*Across*). This design was also used in Experiments 2 and 3, in which audio and visual contexts were presented simultaneously or separately (Experiment 2) or at regular or irregular intervals (Experiment 3). **B** Audio spectrograms of the six soundscapes. Memory for the presented pictures was assessed using a visual object recognition task (**C**) and a temporal order memory task (**D**)

Participants saw 12 lists of 36 visual items, which were randomly selected for each participant from a publicly available image set (Kovalenko et al., 2012). Each item was presented for 2.5 s, with a 2 s interval between consecutive items. Items were presented on an audio-visual background that comprised audioscapes of continuous ambient sounds (audio context) and a colored frame (visual context; Fig. 1). To motivate active encoding of the stimuli (Sheldon, 2020), participants considered for each item how pleasant they found its combination with the audio and the visual contexts (that is, including both the ambient sound and the frame color) during the time the item was presented on the screen. For each list, the audio and visual contexts changed at different rates. In half of the lists, the audio context changed every six items while the visual context changed every nine items. This resulted in two synchronous and six asynchronous audio-visual contextual changes. The asynchronous changes comprised four audio changes (while visual context did not change) and two visual changes (while audio context did not change). In the other half of the lists, the ratio of visual to audio changes was reversed. Note that all contextual changes were multisensory, and that multisensory contexts were continuously presented throughout the encoding of the items.

For each list, frame color was randomly selected from a color set (red, yellow, blue, green, purple, black, white, pink) without repetition. The audioscapes were generated from the soundtracks of publicly available online ambience videos and were chosen to be perceptually different from one another in terms of spectral distribution (Fig. 1B) and perceived environment, and to contain no intelligible speech or resemblance of music. The selected soundtracks included continuous natural sounds from different environments (oceanside beach sounds, cave water drops and rustling, fireplace crackle, office buzz, rainfall and underwater bubbling). The ambient samples were individually edited to match volume, while different frequencies were pronounced in each soundtrack to exaggerate the perceived differences. Sound samples were also stereo separated and minor reverb was added to increase the feeling of immersion. Processed audiofiles were cut to 41 s duration.

After each list, item recognition and temporal order memory were tested in two separate tasks. In the recognition task, participants were presented with items that were either shown during the preceding list or not and had to indicate whether they thought they saw it during the preceding list (Old judgment) or not (New judgment). Items that were drawn from the preceding list were taken directly after a contextual change (boundary item) or three items away from a contextual change, provided that it did not overlap with a following contextual change (non-boundary item; see Fig. 1A). Lure items were drawn from a set of items that were never presented during any of the encoding lists. Each recognition test trial ended upon button response, with a maximum response window of 6 s.

In the temporal order memory test, participants saw pairs of items that were drawn from the preceding list and had to indicate which item of the pair was presented first. The items of a temporal order pair were presented on the left and right side of the center of the screen. The item that was presented first during the encoding phase was shown on the left in half of the trials, and shown on the right in the other half of the trials. The order of trials with left or right-sided presentation of the first encoded item was randomized across lists and participants. Pairs were either drawn from the same audiovisual context (*Within* pair) or from opposite sides of a contextual change (*Across* pair), with the number of items spanning between those of the pair being equal for *Within* and *Across* trials. Further, *Across* trials included pairs that were drawn across synchronous or asynchronous audiovisual context changes. Each trial ended upon button response, with a maximum response window of 6 s.

### Analysis

For the recognition task, we calculated d’ from the hit rates and false alarm rates (Macmillan & Creelman, 2005; Stanislaw & Todorov, 1999) for each of the three types of boundary (non-boundary, asynchronous boundary and synchronous boundary). We then calculated statistical effects using a one-way repeated measures ANOVA with Boundary as within-subject factors. Average response times for correct trials were also analysed using the same ANOVA model.

For the temporal order task, we calculated hit rate for the three types of context (within the same context, across asynchronous contexts and across synchronous contexts) and calculated statistical effects using a one-way repeated measures ANOVA with Context as within-subject factors. The same ANOVA model was used for analysis of the response times (correct trials only).

All ANOVA models included a full-factorial interaction term, using Type III sum of squares. Post hoc paired comparisons were conducted to parse significant main or interaction effects (*p* < 0.05). We report partial eta-squared, ${\eta }_{p}^{2}$, as effect size for significant ANOVA main or interaction effects, unless otherwise stated. Post hoc comparisons are reported as F-tests and ${\eta }_{p}^{2}$ to facilitate comparison with ANOVA outcomes. All statistical analyses were conducted using the open-source freeware package JASP (JASP Team, 2018), which runs on all three major operating systems.

## Results

### Recognition task

**Fig. 2:**
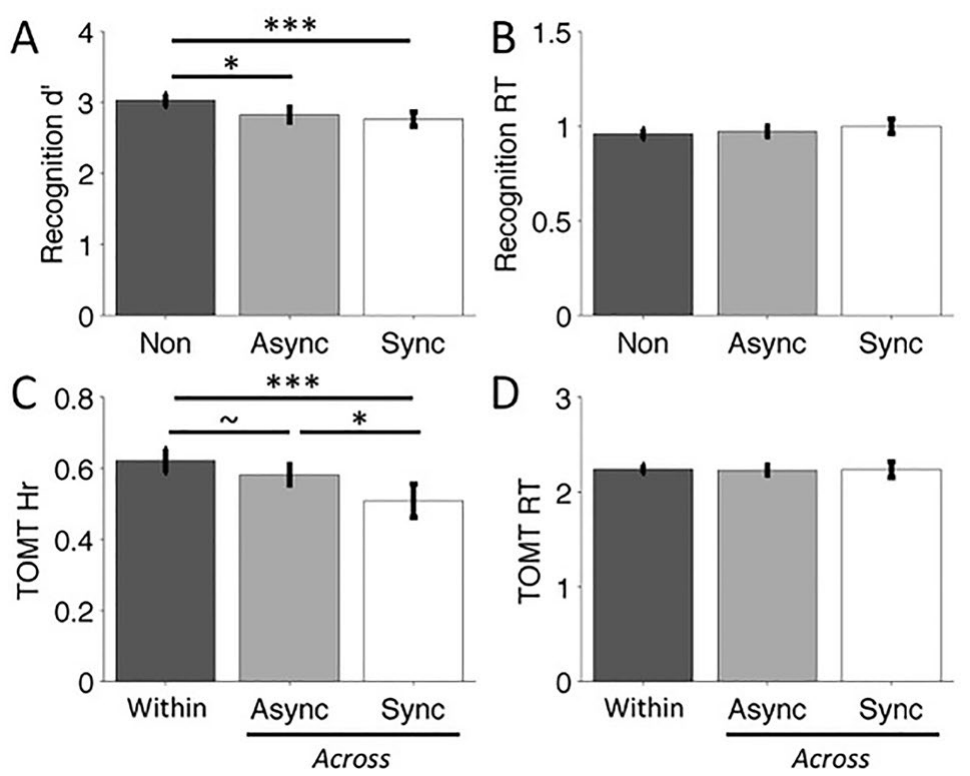
Results of Experiment 1. **A**, **B** Mean d’ (**A**) and response time (RT) in seconds (**B**) for non-boundary (Non), asynchronous boundary (Async) and synchronous boundary (Sync) items in the recognition task. **C**, **D** Mean hit rate (**C**) and RT in seconds (**D**) for the Within- and Across-context conditions of the temporal order memory task (TOMT). Error bars indicate 95% confidence intervals (Masson & Loftus, 2003). *p* < 0.1, *p < 0.05, ***p < 0.005

Performance on the recognition task was relatively high across the three boundary types, with recognition accuracy around 0.9 and false alarm rates around 0.1 (see Table 1 for average hit and false alarm rates per condition). Figure 2A shows the means and 95% confidence intervals of d’ for each of the three boundary conditions. A one-way repeated measures ANOVA revealed a significant effect of Boundary (F(2,46) = 5.44, *P* = 0.0076, ${\eta }_{p}^{2}$=0.19), with significantly higher performance for non-boundary items (mean ± SE *d*’ = 3.00 ± 0.17) compared to asynchronous boundary items (2.78 ± 0.19; *F*(1,23) = 7.21, *P* = 0.013, ${\eta }_{p}^{2}$=0.24) and synchronous boundary items (2.75 ± 0.16; *F*(1,23) = 9.99, *P* = 0.004, ${\eta }_{p}^{2}$=0.30). Performance for the asynchronous boundaries did not significantly differ from the synchronous boundaries (*F*(1,23) = 0.10, *P* = 0.76).

**Table 1:** Mean (SE) hit and false alarm rates of the recognition tests for all three experiments

Exp	AV	Pos	HR		FAR	
			M	SE	M	SE
1	AV	Within	0.92	0.01	0.10	0.02
	AV	Async	0.90	0.02	0.14	0.02
	AV	Sync	0.86	0.02	0.10	0.02
2	A	P1	0.92	0.01	0.08	0.01
		P4	0.94	0.01	0.07	0.01
	V	P1	0.91	0.02	0.06	0.01
		P4	0.94	0.01	0.08	0.02
	AV	P1	0.95	0.01	0.06	0.01
		P4	0.93	0.01	0.08	0.01
3	A	P1	0.86	0.04	0.10	0.03
		P4	0.89	0.02	0.08	0.01
	V	P1	0.90	0.03	0.09	0.02
		P4	0.91	0.02	0.09	0.02
	A	P1	0.88	0.04	0.10	0.02
		P4	0.89	0.03	0.08	0.02
	V	P1	0.89	0.03	0.10	0.02
		P4	0.89	0.02	0.08	0.01

*HR* hit rate, *FAR* false alarm rate, *Exp* experiment, *AV* audio-visual context

The asynchronous boundary trials included two types of unisensory contextual changes: audio but no visual changes and visual but no audio changes. To explore whether these two asynchronous boundaries affected recognition performance differently, we conducted a post hoc paired samples T test and found no significant effect (*F*(1,23) = 1.92, *P* = 0.18).

Figure 2B shows the means and 95% confidence intervals of response times (correct trials only) for each of the three boundary items. Repeated measures ANOVA of recognition response times yielded no significant effect of Boundary (*F*(2,46) = 1.60, *P* = 0.21), with comparable average response times for each of the three boundary items (mean ± SE non-boundary = 963.96 ± 26.54 ms; asynchronous boundary = 971.08 ± 29.29 ms; synchronous boundary = 1008.53 ± 42.19 ms). Post hoc comparison between the two asynchronous boundary items yielded no significant difference (*F*(1,23) = 1.92, *P* = 0.18).

### Temporal order task

Figure 2C shows mean hit rate and 95% confidence intervals for each of the three contexts. A one-way repeated measures ANOVA yielded a significant effect of Context (*F*(2,46) = 8.97, *P* < 0.001, ${\eta }_{p}^{2}$=0.28). Temporal order judgments for pairs crossing a synchronous boundary (mean ± SE = 0.50 ± 0.02) were significantly less accurate than judgments for pairs crossing an asynchronous boundary (0.58 ± 0.02; *F*(1,23) = 2.68, *P* = 0.013, ${\eta }_{p}^{2}$=0.24), as well as for judgments for pairs drawn from the same audiovisual context (Within Context pairs; 0.62 ± 0.02; *F*(1,23) = 12.11, *P* = 0.002, ${\eta }_{p}^{2}$=0.34). The lower accuracy for temporal order judgments from asynchronous Across compared to Within context pairs did not reach significance (*F*(1,23) = 3.61, *P* = 0.07, ${\eta }_{p}^{2}$=0.14). Post hoc comparison between temporal order judgments crossing the two types of asynchronous boundary yielded no significant effect (*F*(1,23) = 1.86, *P* = 0.19).

Figure 2D shows the means and 95% confidence intervals of response times (correct trials only) for each of the three temporal order contexts. Repeated measures ANOVA of recognition response times yielded no significant effect of Context (*F*(2,46) = 0.02, *P* = 0.98), with comparable average response times for each of the three boundary conditions (mean ± SE No Boundary = 2010.59 ± 103.60 ms; asynchronous boundary = 2016.10 ± 107.35 ms; synchronous boundary = 2025.53 ± 135.83 ms). Post hoc comparison between the two asynchronous boundary types yielded no significant difference (*F*(1,23) = 0.29, *P* = 0.60).

## Discussion

We observed two main findings. First, we found that recognition memory was worse for items that were presented at multisensory boundaries compared to non-boundary items, regardless of multisensory synchrony. This finding suggests that synchronous boundaries affected recognition memory in a similar way as asynchronous boundaries. The superior performance for non-boundary items contrasts findings from previous movie segmentation studies (Meitz et al., 2020; Schwan & Garsoffky, 2004; Schwan et al., 2000; Swallow et al., 2009), in which filmic information at boundaries is commonly better recognized than non-boundary information. Interestingly, studies using a similar segmentation task as ours, in which series of face pictures were interspersed with a semantic boundary of visual objects (or vice versa) (DuBrow & Davachi, 2013, 2014), reported recognition performance (hit and false alarm rates) comparable to our results, with no significant difference between boundary and non-boundary items. These results, in combination with ours, could indicate that the effect of contextual boundaries on recognition performance of picture series may defer from recognition of movie stimuli.

Second, we found that multisensory synchronicity of contextual changes affected temporal order memory, with synchronous boundaries impairing temporal associative processing more than did asynchronous boundaries. This finding supports the suggestion that synchronous multisensory boundaries are better processed than asynchronous changes, which, in keeping with the proposed encoding-memory trade-off, comes at a greater expense of temporal memory interference (Heusser et al., 2018). Synchronous multisensory boundaries may constitute a higher sensory dimensionality that may more likely lead to event model updating. This reasoning is in line with previous findings that reading times of narrative texts increased at boundaries of higher narrative dimensionality, e.g., co-occurring changes in time, space or perspective (Meyerhoff & Huff, 2016; Radvansky & Copeland, 2010).

A previous study found that event perception and memory of audiovisual movie clips did not differ between synchronous and asynchronous audio-visual tracks (Meyerhoff & Huff, 2016). The authors reasoned that participants segmented the audiovisual clips in all conditions similarly because the audio and visual manipulations did not alter the semantic predictions derived from the clips. That is, the semantic overlap between the audio and visual tracks made mismatching sensory-level information redundant in event segmentation. In our experiment, the pictures were not conceptually related to the visual or audio contexts, suggesting that segmentation involved expectations at sensory rather than semantic levels.

A limitation of Experiment 1 was that all boundaries constituted multisensory changes. Therefore, we could not investigate whether asynchronous multisensory contexts affected memory differently from truly unisensory contexts, in which only one sensory modality would provide contextual boundaries in the complete absence of the other modality. Further, as Experiment 1 is, to our knowledge, the first to use arbitrary audio and visual contexts to induce event boundaries, the recognition and temporal order memory effects require conceptual replication. These two issues were addressed in Experiment 2.

## Experiment 2

Experiment 2 was designed to test if (synchronous) multisensory boundaries affected memory performance more than unisensory boundaries. Importantly, in contrast to Experiment 1, unisensory boundaries in Experiment 2 constituted contextual changes in one sensory context while the other context was entirely absent. The outcome of this experiment could further elucidate the results of Experiment 1. Finding a larger temporal memory impairment for multisensory as opposed to unisensory boundaries would indicate that the effect of a boundary on memory increases with increasing perceptual dimensionality of the contextual changes. This finding would then suggest that the results of Experiment 1 arose from increased perceptual dimensionality of the synchronous multisensory boundaries. However, finding that uni- and multi-sensory boundaries similarly affected temporal order memory would indicate that perceptual dimensionality per se cannot explain the results of Experiment 1.

## Methods

### Participants

We recruited 20 new participants (9 females; mean ± SD age = 22.4 ± 1.6 years, range 20–26) from the same academic environment and using the same procedures. All participants gave informed consent before participating in the experiment and were monetarily compensated. The study was approved by the ethical committee of the Faculty of Psychology and Neuroscience of Maastricht University.

### Procedures

**Fig. 3:**
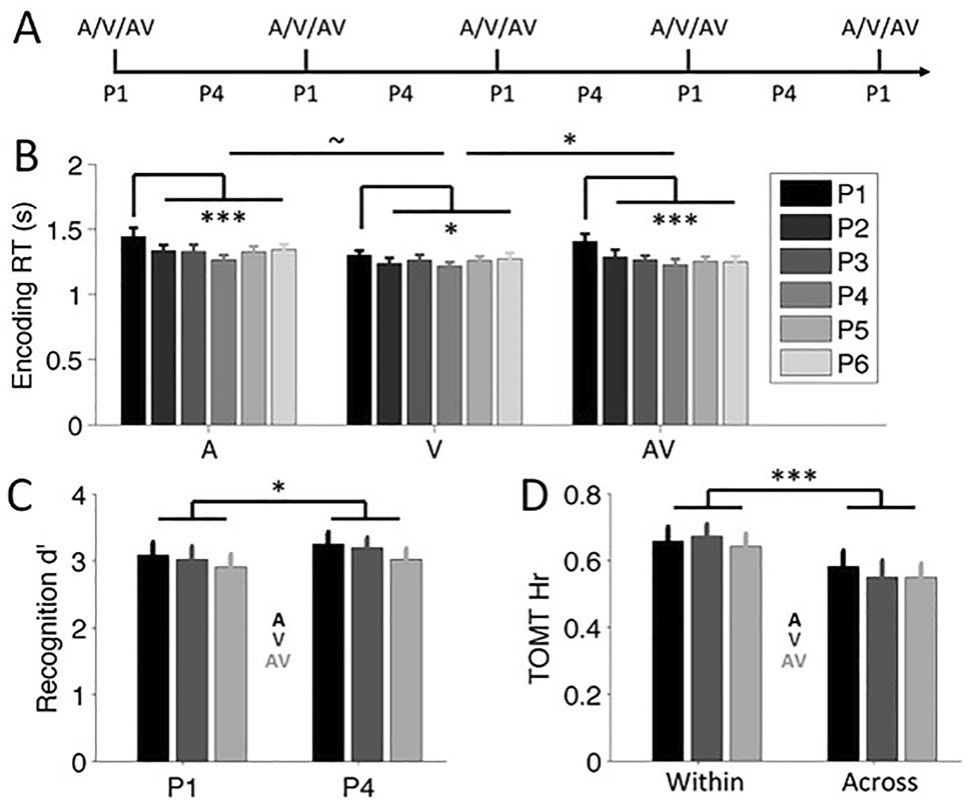
Design and results of Experiment 2. **A** Experiment 2 included unisensory audio (A) and visual (V) boundaries, and multisensory audiovisual (AV) boundaries. **B** Encoding response time (RT) for the boundary item (P1) was significantly slower than encoding time for non-boundary items at subsequent positions. Relatively slower boundary item response time was larger for AV compared to V context. **C** Pooled across contexts (audio in black, visual in dark gray, audiovisual in light gray), recognition sensitivity (d’) was higher for the middle non-boundary item (P4) compared to the boundary item (P1). **D** Pooled across contexts, temporal order memory accuracy (hit rate, Hr) for Within-context judgments was higher than for Across-context judgments. Error bars represent 95% confidence intervals (Masson & Loftus, 2003). *p* < 0.1, *p < 0.05, ***p < 0.005

We used a similar design as Experiment 1, with the following changes. First, pictures were presented in one of three contexts: audio only (*A*, playing the soundscapes without any frame color), visual only (*V*, changing frame color without playing any soundscape) or audio-visual (*AV*, simultaneous presentation of soundscapes and colored frames). Second, in each context condition, a context changed after every six pictures. Further, in the audio-visual context, the frame changed color simultaneously with a change in soundscape. Figure 3A shows a schematic representation of the design of Experiment 2.

Previous studies found that contextual changes resulted in slower response times during encoding (Heusser et al., 2018; Radvansky & Copeland, 2010; Zacks et al., 2009), possibly due to the increased load that event model updating puts on working memory processing (Zacks, 2020). To assess whether multisensory boundaries taxed encoding processes more than unisensory boundaries, we logged and analysed encoding response times in Experiment 2. At the start of each list, participants were instructed to overtly report their perceived pleasantness about the combination of each visual item and the audio, visual or audio-visual context by pressing either key “1” (“Pleasant”) or key “2” (“Unpleasant”) on the computer keyboard while the respective item was available on the screen. The item did not disappear after key press, so that presentation time remained the same for all items.

## Results

### Encoding

Encoding responses of one participant were not properly logged, leaving 19 datasets for the analysis of encoding responses. Figure 3B shows the mean encoding time for all six positions in the three contexts of the remaining 19 participants. To test whether boundary item responses were slower than non-boundary item responses, we averaged response times across non-boundary items for each context and calculated a repeated measures ANOVA with Position (boundary vs. non-boundary positions) and Context (audio, visual, audio-visual) as within-subject factors. This analysis yielded significant main effects for Position (*F*(1,18) = 19.57, *P* < 0.001, ${\eta }_{p}^{2}$=0.52) and Context (*F*(2,36) = 11.42, *P* < 0.001, ${\eta }_{p}^{2}$=0.39), and a significant Position × Context interaction effect (*F*(2,36) = 4.41, *P* = 0.019, ${\eta }_{p}^{2}$=0.20). Post hoc comparisons showed significantly slower response times for boundary items compared to non-boundary items for the audio (*F*(1,18) = 10.40, *P* = 0.005, ${\eta }_{p}^{2}$=0.37), visual (*F*(1,18) = 6.97, *P* = 0.017, ${\eta }_{p}^{2}$=0.28) and audiovisual contexts (*F*(1,18) = 19.63, *P* < 0.001, ${\eta }_{p}^{2}$=0.52).

The significant Position × Context interaction effect suggested that the slower encoding time associated to boundary items differed between contexts. To assess whether this was the case, we used post hoc comparisons to compare the slower boundary response times relative to the non-boundary response times between the various uni- and multi-sensory contexts. The relatively slower boundary response times (compared to non-boundary response times) for visual context was marginally significantly smaller than for auditory context (V *minus* A: *F*(1,18) = 3.26, *P* = 0.088, ${\eta }_{p}^{2}$=0.15) and significantly smaller than for audiovisual context (V *minus* AV: *F*(1,18) = 7.48, *P* = 0.014, ${\eta }_{p}^{2}$=0.29). Relatively slower boundary response times did not differ between auditory and audiovisual contexts (A *minus* AV: *F*(1,18) = 1.06, *P* = 0.32). Thus, audio and audiovisual boundaries led to slower boundary encoding times than did visual boundaries.

Could this difference in response slowing be related to increased difficulty in rating audio contexts (uni- or multi-sensory) over visual contexts? To address this issue, we conducted a post hoc one-way repeated measures ANOVA on the non-boundary encoding times of the three sensory contexts. We found no significant effect (*F*(2,56) = 0.34, *P* = 0.99), suggesting that difficulty of non-boundary judgments was comparable between the uni- and multi-sensory contexts.

### Recognition

As in Experiment 1, recognition accuracy was again relatively high, with accuracy around 0.9 for both boundary and non-boundary positions in the auditory, visual and audio-visual contexts. False alarm rates were below 0.1 across all conditions (see Table 1 for average hit and false alarm rates across all conditions). Figure 3C shows mean sensitivity (d’) for each condition, with the 95% confidence interval plotted as error bars. A two-way repeated measures ANOVA with Position (P1, P4) and Context (audio, visual, audiovisual) as within-subject factors yielded a significant effect for Position (*F*(1,19) = 4.46, *P* = 0.048, ${\eta }_{p}^{2}$ = 0.19), with higher recognition for middle items (P4 items, pooled across contexts; mean ± SE *d*’ = 3.2 ± 0.14) compared to boundary items (P1 items, 3.0 ± 0.15). We found no significant main effect for Context (*P* = 0.15) or the Position × Context interaction term (*P* > 0.9).

A two-way repeated measures ANOVA for recognition response times revealed a significant effect for Position (*F*(1,19) = 9.35, *P* = 0.006, ${\eta }_{p}^{2}$=0.33), with faster response times for middle items (1117.6 ± 45.8 ms) compared to boundary items (1189.0 ± 55.5 ms). We found no significant effect for Context (*P* = 0.45) or the Position × Context interaction (*P* = 0.42). These findings suggest better performance for middle items compared to boundary items, in line with the recognition accuracy results.

### Temporal order memory

Figure 3D shows mean hit rate for temporal order memory in each context, with the 95% confidence interval plotted as error bars. The two-way repeated measures ANOVA with Event (within, across) and Context (audio, visual, audiovisual) as within-subject factors yielded a significant effect for Event (*F*(1,19) = 20.59, *P* < 0.001, ${\eta }_{p}^{2}$=0.52), but no significant effect for Context (*F*(1,19) = 0.75, *P* = 0.48) or the Event × Context interaction term (*F*(2,38) = 0.66, *P* = 0.53). Post hoc comparisons showed that hit rates for *Within* trials were significantly higher than those for the *Across* trials of all three sensory contexts (see Table 2).

**Table 2:** Temporal order memory results of Experiment 2

	Within		Across				
	M	SE	M	SE	T	P	Cd
A	0.66	0.03	0.58	0.02	2.35	0.03	0.52
V	0.67	0.02	0.55	0.03	3.94	< 0.001	0.88
AV	0.64	0.03	0.55	0.02	2.81	0.011	0.63

Mean (SE) of Within and Across temporal order memory trials and post hoc comparison statistics (degrees of freedom = 19)

*A* audio, *V* visual, *AV* audiovisual, *Cd* Cohen’s d

Experiment 1 showed that temporal order accuracy differed for item pairs crossing the synchronous and asynchronous boundaries. To test for a similar effect in Experiment 2, we first conducted a one-way repeated measures ANOVA of the *Across* trials, with Context (audio, visual, audiovisual) as within-subject factor. The main effect of Context was not significant (*F* < 1). To mimick the asynchronous condition of Experiment 1, we pooled the *Across* trials of the two unisensory conditions of Experiment 2 and compared it to the *Across* trials of the multisensory condition (one-way repeated measures ANOVA). We again found no significant effect (*F* < 1), suggesting that uni- and multi-sensory boundaries in Experiment 2 similarly affected temporal order accuracy.

Finally, a repeated measures ANOVA of the temporal order memory response times showed a significant effect for Event (*F*(1,19) = 8.37, *P* = 0.009, ${\eta }_{p}^{2}$=0.31), with faster response times for *Within* trials (2445.1 ± 0.13 ms) than *Across* trials (2618.4 ± 0.12 ms). There was no significant effect for Context (*P* = 0.17) or the Event × Context interaction (*P* = 0.12).

## Discussion

Our finding of slower encoding times for boundary compared to non-boundary items replicated previous findings in unisensory segmentation (Zacks et al., 2009; Radvansky & Copeland, 2010; Heusser et al., 2018; Huff et al., 2018; van de Ven et al., 2021). However, we also found that audio-related boundaries affected encoding more than (uni-sensory) visual boundaries, which was not due to increased overall difficulty in rating audio contexts. One explaining factor is that changes in audio contexts take time to be processed, as the soundscapes are perceptually defined over time, while the color change in the visual context is instantaneous.

Despite this modality-dependent difference in perceptual identification, we did not find evidence for sensory dimensionality (i.e., uni- vs. multi-sensory contextual changes) affecting perceptual boundary processing. That is, synchronous multisensory contextual changes were processed in a similar way as unisensory contextual changes. This finding is in agreement with a previous multisensory segmentation study of movie clips (Meitz et al., 2020), in which participants detected across-scene boundaries better than within-scene boundaries regardless of whether the audio track was audible. More generally, our finding may weigh in on the inconsistent results of encoding time of boundary items increasing (Zacks et al., 2009) or not changing with higher dimensional complexity [Experiment 3 in (Huff et al., 2018)], with our findings supporting the latter. In another study, reading times during story reading were analysed when contextual changes occurred in one or more narrative dimensions, such as spatial, temporal, goal-directed and protagonist-related contexts (Zwaan et al., 1998). Results showed that reading times were systematically slower for non-spatial contextual changes. However, reading times for spatial contextual changes only slowed when participants had learned the spatial environment of the story prior to reading it, regardless of whether the spatial changes were clearly demarcated or not. The authors suggested that the familiarization prior to reading could have made the spatial context more relevant to understanding the story, thereby enhancing its role in segmentation. In our Experiment 1, the synchronous multisensory changes may have become more relevant when offset to the asynchronous changes, while in Experiment 2, the multisensory changes offered no new boundary information with respect to the unisensory changes.

In the memory domain, we again found better recognition memory for non-boundary compared to boundary items, thus replicating our finding in Experiment 1. Further, we found no recognition difference between uni- and multi-sensory contexts, which contrasts previous findings of better multisensory than unisensory recognition of movie clips (Meitz et al., 2020; Meyerhoff & Huff, 2016). However, an important difference with these studies is that in our task, the items were not semantically related to the contextual information, allowing separation of context-induced boundary processing from item memory.

Finally, we found worse temporal memory performance for items crossing a boundary compared to items from the same context, regardless of sensory modality or complexity of the boundary or context. This finding fits with the suggestion that contextual boundaries impair temporal associative processing similarly for different sensory modalities or complexity. In sum, these findings suggest that synchronous multisensory boundaries affect memory in similar ways as unisensory boundaries. By extension, the effect of synchronous multisensory boundaries on temporal memory in Experiment 1 does not seem to be the result of multisensory dimensionality per se.

An alternative explanation could be that boundary processing in Experiment 1 was augmented by the uncertainty about when a boundary would occur, due to the mix of synchronous and asynchronous boundaries during a list. Previous studies have shown that rhythmic stimulus presentation enhances attentional processing, compared to non-rythmic presentation (Rohenkohl et al., 2011; Jones & Ward, 2019; ten Oever & Sack, 2019). In Experiment 1, the decreased predictability of boundary occurrence could have impaired the temporal deployment of attention, resulting in less efficient processing of the asynchronous boundaries and subsequently less impaired temporal order memory. In Experiment 2, the regular occurrence of both uni- and multi-sensory boundaries could have led to comparable boundary effects on temporal order memory, thereby obscuring a possible effect of temporal uncertainty on boundary processing. The possible role of boundary predictability was investigated in Experiment 2.

## Experiment 3

In Experiment 3, unisensory audio or visual contexts changed regularly or irregularly during encoding, such that boundary occurrence was temporally predictable or unpredictable, respectively. We included only the two unisensory contexts to maximize the statistical power of the design, as Experiment 2 indicated no explanatory power of synchronous multisensory over unisensory boundaries. Finding a larger temporal memory impairment for regular boundaries, compared to non-regular boundaries, would indicate that temporal expectancy about when contextual changes will occur affects encoding and memory formation. It would then explain the results of Experiment 1 by the temporal irregularity of the occurrence of the synchronous and asynchronous boundaries. However, finding comparable effects of regular and irregular boundaries would suggest that temporal expectancy about boundary occurrence does not modulate temporal segmentation in perception and memory.

## Methods

### Participants

We recruited 20 new participants (8 females; mean ± SD age = 22.6 ± 2.2 years, range 20–26) from the same academic environment. Recruitment and exclusion criteria were the same as for Experiment 1. All participants gave informed consent before participating in the experiment and were monetarily compensated. The study was approved by the ethical committee of the Faculty of Psychology and Neuroscience of Maastricht University.

### Procedure

**Fig. 4:**
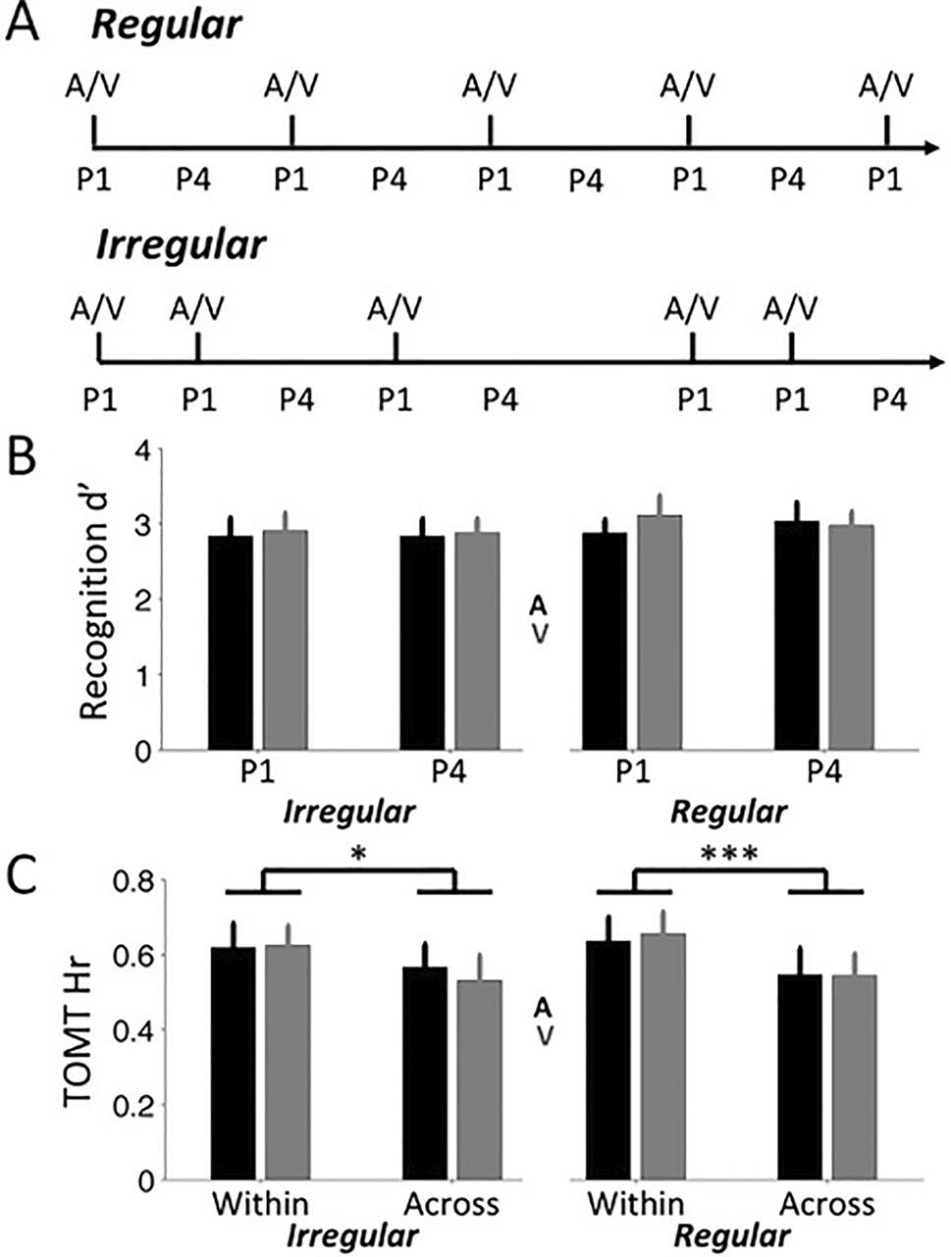
Design and results of Experiment 3. **A** Audio or visual boundaries were presented regularly or irregularly throughout a list of pictures. Recognition memory sensitivity (**B**) and temporal order accuracy (**C**) for the regular and irregular conditions (audio boundaries in black and visual boundaries in gray bars)

The experiment instructions and procedure were the same as in Experiment 2, including the overt encoding responses, the recognition and the temporal order memory tasks, but with the following exceptions. In Experiment 3, we only used the unisensory contextual conditions. Participants completed six visual-only and six audio-only context lists. In each unisensory condition, the contextual changes either occurred consistently and regularly after the 6^th^ image (regular condition), or occurred irregularly after three, six or nine objects (irregular condition). Figure 4A depicts the temporal structure of unisensory contextual changes in the regular and irregular conditions. In both conditions, the visual or audio context changed five times. The order of contextual intervals in the irregular condition varied across different lists. At the start of each list, the participant saw a cue specifying whether the contextual changes of the list would be regular or irregular. The order of lists with visual or audio contexts, and regular or irregular context changes was randomized for each participant.

## Results

### Encoding

Encoding response times were incomplete or missing for eight participants, possibly due to these participants accidentally pressing the wrong keys during encoding. To assess the effect of regularity of the contextual changes on encoding response times, we compared response times between the boundary and (the pooled) non-boundary items. A repeated measures ANOVA with Position (boundary, non-boundary), Context (A, V) and Regularity as within-subject factors yielded a significant main effect of Position (*F*(1,11) = 40.28, *P* < 0.001, ${\eta }_{p}^{2}$=0.79) and of Context (*F*(1,11) = 9.27, *P* = 0.011, ${\eta }_{p}^{2}$=0.46), and a significant Position × Context interaction term (*F*(1,11) = 7.66, *P* = 0.018, ${\eta }_{p}^{2}$=0.41). The effects related to Context resulted from slower boundary response times for audio contextual changes, compared to visual contextual changes, in the Irregular (*F*(1,11) = 7.98, *P* = 0.017, ${\eta }_{p}^{2}$=0.42) and the Regular condition (*F*(1,11) = 4.22, *P* = 0.065, ${\eta }_{p}^{2}$=0.28). More importantly, none of the effects related to Regularity were significant (main effect *P* > 0.9, first-order interaction effect Ps > 0.6, second-order interaction effect *P* = 0.23). Thus, the slower encoding times for boundary items did not differ between regular and irregular contextual changes.

### Recognition

As in Experiments 1 and 2, recognition performance was relatively high, with overall hit rate around 0.9 and overall false alarm rate around 0.1 (see Table 1 for average hit and false alarm rates across all conditions). Figure 4B shows recognition sensitivity (d’) for the boundary (P1) and non-boundary items (P4) in the regular and irregular unisensory conditions. A repeated measures ANOVA with Position, Context and Regularity as within-subject factors revealed no significant main or interaction effects (all Ps > 0.20). Thus, neither regularity nor modality of the contextual changes affected recognition performance.

Analysis of response times (correct responses only) using a similar repeated measures ANOVA model yielded no significant main effects (Ps > 0.31) or interaction effects (Ps > 0.40). Average ± SE response times across all conditions was 1084.17 ± 45.67 ms, comparable to that of the previous experiments.

### Temporal order memory

Figure 4C shows the performance accuracy on the temporal order memory test. Accuracy for within-context temporal order judgments was higher than for across-context judgments for both audio and visual contextual changes. A repeated measures ANOVA with Event (within, across), Context and Regularity revealed a significant main effect of Event (*F*(1,18) = 14.48, *P* = 0.0013, ${\eta }_{p}^{2}$=0.45), thus statistically supporting superior within-context performance. Other main or interaction effects were not significant (Ps > 0.36).

To assess whether irregular unisensory boundaries affected temporal order memory differently than regular boundaries, *Across* trial data was pooled across the audio and visual modalities and analysed using a one-way repeated measures ANOVA with Regularity as within-subject factor. We found no significant effect (*F* < 1).

Analysis of responses times (correct responses only) using a similar repeated measures ANOVA model yielded no significant main (Ps > 0.14) or interaction effect (Ps > 0.26). Average ± SE response times across all conditions was 2477.14 ± 183.59 ms, which was also comparable to the previous experiments.

## Discussion

We found no evidence that temporal irregularity of boundary occurrence affected recognition or temporal memory, which suggests that temporal irregularity did not modulate boundary processing in terms of event model updating (Zacks, 2020) or encoding instability (Clewett & Davachi, 2017). Temporal segmentation may depend on the occurrence or presence of a contextual boundary, rather than the temporal regularity by which they occur. Further, these findings suggest that temporal irregularity does not explain the synchronous multisensory boundary effect of Experiment 1.

## General discussion

Our findings can be summarized as follows. Synchronous multisensory contextual changes during encoding interrupted temporal associative memory processing more than asynchronous multisensory changes. This effect could not be explained by higher sensory dimensionality of multisensory over unisensory contexts, nor by the temporal irregularity of boundary occurrence during encoding. Instead, we argue that the synchronous multisensory boundaries constituted a stronger deviation from multisensory expectations than asynchronous boundaries. Further, the effect of multisensory synchronicity was not found in recognition memory, similarly to a previous multisensory segmentation study, in which asynchronous audio and visual tracks of movie clips did not alter memory performance with respect to synchronous movie clips (Meyerhoff & Huff, 2016). Our findings underscore the suggestion that temporal associative memory tests may be more sensitive to the effect of contextual boundaries on memory formation than recognition memory (Clewett & Davachi, 2017; Heusser et al., 2018). Notably, in Meitz et al., movie clip asynchrony was obtained by playing the visual track in reverse. This approach differs from our implementation of multisensory asynchrony by temporal onset difference, which is more comparable with implementations in multisensory integration research [e.g., (Van Atteveldt et al., 2007; Chen & Spence, 2010; ten Oever et al., 2013)]. Reversing the visual track may have biased semantic processing over perceptual processing in segmentation, whereas our paradigm favoured perceptual processing in the absence of semantic relatedness.

The results of Experiment 2 indicated that, in the absence of asynchronous boundaries, the unisensory and multisensory boundaries affected encoding and memory in a similar way. This result appears at odds with findings that multisensory stimuli are better encoded or remembered than unisensory stimuli (Botta et al., 2011; Thompson & Paivio, 1994). However, in such studies, participants had to detect or memorize the multisensory items themselves, whereas in our case, participants did not have to detect or remember the uni- or multi-sensory backgrounds. Further, the audio and visual contexts were not semantically related to the encoded pictures, thereby limiting the interaction between picture encoding and uni- or multi-sensory background features.

In Experiment 3, the finding that memory did not depend on the temporal regularity of boundaries aligns with event segmentation models that propose that a contextual boundary violates expectations of the currently active event model in working memory (Radvansky & Zacks, 2017; Zacks, 2020). From this perspective, the irregularity of boundary occurrence across events will not violate expectations of individual events, unless temporal context is a defining feature of those events (van de Ven et al., 2021). Further evidence for this notion comes from segmentation studies using naturalistic stimuli (Meyerhoff & Huff, 2016; Schwan & Garsoffky, 2004; Sridharan et al., 2007; Zacks et al., 2009), in which participants segment those stimuli despite a substantial variation in scene length that causes temporal irregularity of the contextual boundaries.

The results provide further insight into how contextual changes lead to temporal segmentation. Several theories have proposed that segmentation depends on event models that provide situational predictions, which are derived from the temporal integration of previous experiences or prior semantic or schematic knowledge (Radvansky & Zacks, 2017; Richmond & Zacks, 2017; Zacks, 2020). A perceived contextual change violates event-based predictions, which triggers prediction error and prompts updating of the event model in working memory to better accommodate the new context. Prediction error in segmentation is processed in prefrontal and striatal areas (Sridharan et al., 2007; Zacks et al., 2011), which also process prediction error in reward-based learning (Garrison et al., 2013; Gershman & Uchida, 2019). In our experiments, participants could have formed perceptual expectations about the multisensory backgrounds, for which the synchronous multisensory boundaries posed the largest deviation from those expectations. However, it is unclear if violations of perceptual expectations would elicit prediction error in working memory or reward processing areas. The lack of semantic relation between pictures and background features argue against a prominent role of event-level prediction error. Other studies have also found that changes in semantically unrelated contextual features during encoding, such as frame color (Heusser et al., 2018) or timing (Logie & Donaldson, 2021; van de Ven et al., 2021), affect memory formation. These findings arguably better fit to the alternative suggestion that segmentation is based on encoding instability that arises from changing contextual features without a conceptual event model (Clewett & Davachi, 2017). In this view, uni- or multi-sensory boundaries could segment information in memory without requiring event-level prediction error in working memory. However, an unresolved issue in this view is that the monitoring of encoding stability requires some comparison between current and previous perceptual states, which may re-introduce prediction error-like processing (de Lange et al., 2018; Keller & Mrsic-Flogel, 2018), albeit for perceptual rather than conceptual features, and therefore in lower level (sensory) brain areas rather than working memory.

An important consideration is that we implicitly inferred boundary processing from performance on encoding or memory tasks, rather than having participants explicitly report on their observed contextual changes. In “unitization tasks”, participants watch a movie or read a narrative and are asked to overtly indicate (e.g., via button press) when they think a meaningful event has ended or a new one has started (Huff et al., 2014; Lassiter & Slaw, 1991; Newtson & Engquist, 1976; Newtson et al., 1977). What is considered “meaningful” is inherently subjective, which can lead to substantial variability in unitization across participants (Sargent et al., 2013). The temporal co-occurrence of a boundary across participants can be regarded as the segmentation magnitude of that boundary, with higher segmentation magnitude indicating that participants tend to show more similar unitization (Huff et al., 2014). Previous studies have suggested that increased contextual dimensionality may increase segmentation magnitude, which in turn may facilitate memory formation (Flores et al., 2017; Huff et al., 2014; Sargent et al., 2013). Extrapolating from our findings, it may be synchronicity of contextual feature changes, rather than dimensionality, influencing segmentation magnitude. However, this notion remains to be tested. Further, it is unknown if unitization for items that lack semantic relatedness, as in our task, is different than for semantically related informations, such as in narratives. Combining the two tasks could reveal new insights in how contextual changes drive segmentation and support memory formation.

A possible limitation of our study is that participants completed the task outside of a controlled laboratory. We aimed to control technical aspects of the study by using freely available and well-tested software that has been verified to run on all major operating systems without major differences in software performance (Bridges et al., 2020). We also provided instructions to limit environmental distractions as much as possible. Further, we replicated previous findings from laboratory settings of slower encoding response times in our design, as well as the typical observation of worse temporal order memory performance for items crossing a boundary. Finally, the high recognition performance in all three experiments suggests that participants effortfully and attentively completed the tasks. We therefore think that our results provide a reliable contribution to the understanding of event segmentation in perception and memory.

In conclusion, we found that the synchronicity of multisensory contextual boundaries affected temporal order memory. Further, neither multisensory dimensionality nor the temporal regularity of boundary occurrence affected temporal or recognition memory. Our findings provide further insight into how contextual changes affect the organization of perceptual and mnemonic processing of our experiences.

## Data Availability

Experimental data and audio stimuli are publicly available via an Open Science Foundation (OSF) project page at https://osf.io/rcx56/.
